# TIA: algorithms for development of identity-linked SNP islands for analysis by massively parallel DNA sequencing

**DOI:** 10.1186/s12859-018-2133-2

**Published:** 2018-04-11

**Authors:** M. Heath Farris, Andrew R. Scott, Pamela A. Texter, Marta Bartlett, Patricia Coleman, David Masters

**Affiliations:** 1 The Homeland Security Systems Engineering and Development Institute (HSSEDI), operated by The MITRE Corporation, McLean, Virginia, USA; 2 The Department of Homeland Security, U.S. Customs and Border Protection, Washington, DC USA; 3 The Department of Homeland Security, Science and Technology Directorate, Washington, DC USA; 4 Department of Advanced Technology, The MITRE Corporation, 7515 Colshire Drive, McLean, Virginia, 22102 USA

**Keywords:** Algorithm, Single nucleotide polymorphism (SNP), Population frequency, Massively parallel sequencing (MPS), Human identity

## Abstract

**Background:**

Single nucleotide polymorphisms (SNPs) located within the human genome have been shown to have utility as markers of identity in the differentiation of DNA from individual contributors. Massively parallel DNA sequencing (MPS) technologies and human genome SNP databases allow for the design of suites of identity-linked target regions, amenable to sequencing in a multiplexed and massively parallel manner. Therefore, tools are needed for leveraging the genotypic information found within SNP databases for the discovery of genomic targets that can be evaluated on MPS platforms.

**Results:**

The SNP island target identification algorithm (TIA) was developed as a user-tunable system to leverage SNP information within databases. Using data within the *1000 Genomes Project* SNP database, human genome regions were identified that contain globally ubiquitous identity-linked SNPs and that were responsive to targeted resequencing on MPS platforms. Algorithmic filters were used to exclude target regions that did not conform to user-tunable SNP island target characteristics. To validate the accuracy of TIA for discovering these identity-linked SNP islands within the human genome, SNP island target regions were amplified from 70 contributor genomic DNA samples using the polymerase chain reaction. Multiplexed amplicons were sequenced using the Illumina MiSeq platform, and the resulting sequences were analyzed for SNP variations. 166 putative identity-linked SNPs were targeted in the identified genomic regions. Of the 309 SNPs that provided discerning power across individual SNP profiles, 74 previously undefined SNPs were identified during evaluation of targets from individual genomes. Overall, DNA samples of 70 individuals were uniquely identified using a subset of the suite of identity-linked SNP islands.

**Conclusions:**

TIA offers a tunable genome search tool for the discovery of targeted genomic regions that are scalable in the population frequency and numbers of SNPs contained within the SNP island regions. It also allows the definition of sequence length and sequence variability of the target region as well as the less variable flanking regions for tailoring to MPS platforms. As shown in this study, TIA can be used to discover identity-linked SNP islands within the human genome, useful for differentiating individuals by targeted resequencing on MPS technologies.

**Electronic supplementary material:**

The online version of this article (10.1186/s12859-018-2133-2) contains supplementary material, which is available to authorized users.

## Background

Single nucleotide polymorphisms, SNPs, are presented as unique point mutations within the genome. As comparative genomics studies increase their fidelity with which the genomes of the human population are examined, the intricacies of the SNP diversity found within the population are described [1], making the detection of population-defining variations more predictable. Definition of these genomic variations provides utility in determining markers of disease states, phenotypic traits, ancestry, and individual identity [2, 3]. The use of SNPs for identity differentiation continues to gain momentum in the forensics community as demonstrated in previously reported SNP panels with forensic applications. For example, the GenPlex HID System [4] utilizes 48 of the 52-plex SNP*for*ID SNP panel, which has a mean probability match of at least 5.0 × 10^− 19^ [5]. Kidd et al. reported a panel of 19 unlinked SNPs [2], a panel of 40 unlinked SNPs [6], and later an expanded panel of 92 unlinked SNPs [7] for application in forensic identification of individuals. In the application of massively parallel sequencing (MPS) technologies in forensic sciences, reports have described the Illumina ForenSeq system [8–11] and the Ion Torrent AmpliSeq HID system [12] for use in human identification and genetic profiling. Each of these use MPS technologies for rapid targeted resequencing analysis of informative SNPs.

Leveraging advances in MPS and post-sequencing bioinformatics processing technologies, SNPs are characterized within the genome with increasing speed and fidelity. Projects such as the *1000 Genomes Project* [13], *International HapMap Project* [14], and the *Encyclopedia of DNA Elements* (ENCODE) [15] offer necessary and valuable databases with SNP representations from the greater global human population. The *1000 Genomes Project* has made available a database containing records of verified SNP locations found in the current version of the human reference genome across 2504 unrelated complete human genomes, representing 26 populations around the world. The SNPs within this database are an informative repository of potential target regions for the development of a suite of genetic markers with utility for determining identity.

In this study, we identified 54 SNP islands that we define as haplotypes of multiple identity-linked SNPs located within the same discrete genomic region. The SNP islands were identified using a state-based computational algorithm filtering through the genome and demonstrated utility for differentiating human identity. SNPs within the islands were selected to provide representation within the human population with a frequency that allows general variability but not niche specificity, avoiding selection of a highly-specific population. The islands were computationally targeted in compact regions of the genome (≤ 400 base pairs in length) containing three to five SNPs with a SNP frequency between 30 and 70% for the global population as defined by data from the *1000 Genomes Project*. Further, each island was required to be flanked by low-variance regions of at least 150 bp, containing only SNPs with frequencies of ≤0.5% or ≥ 99.5%. The identified SNP islands were down-selected for unique genome locations with conserved primer targeting sites within the flanking regions as compared to the whole human genome. These conserved primer sites allow target-specific amplification of the SNP islands while minimizing mis-priming amplicon noise, resulting in increased target sequence resolution. Within the resulting 54 SNP islands, 166 SNP markers for identity passed computational filters, experimental amplification, and massively parallel DNA sequencing. An additional 143 SNPs were identified within the SNP islands. Each of the SNPs provided identity-relevant information, defined as displaying discerning information across individual DNA profiles. Application of the resulting SNP array to contributor genomic DNA produced profiles of the contributing individuals that were targeted, individually specific, and reproducible. Overall, the SNP panel differentiated all 70 individuals that is was tested against. In this report, a subset of this SNP array (27 islands) was used to differentiate 15 individual identity profiles obtained using buccal swab DNA samples (~ 10 ng/μL).

## Methods

### State-based algorithmic SNP island detection

**Fig. 1:**
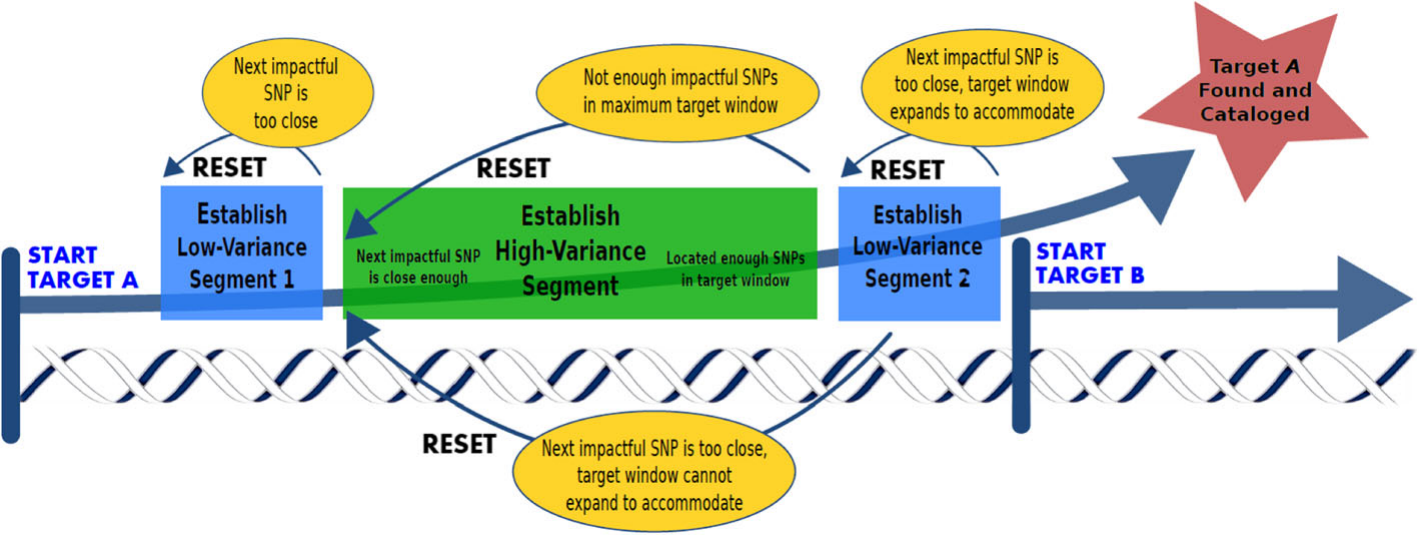
Target Identification Algorithm (TIA) progression through the genome. The algorithm progresses through the genome in a dynamic manner. After the low-variance segment 1 is established, TIA finds the high-variance segment, containing the requisite number of impactful SNPs within the defined sequence length. Once the high-variance segment is established, TIA finds the low-variance segment 2. If the algorithm fails to find the appropriate segment as it progresses through the genome, it resets itself to begin the search again

The SNP island target identification algorithm (TIA; Fig. 1) was developed as a script using Python version 2.6.6 [16] and utilized allele frequency variant call files from the *1000 Genomes Project Database*. The user-defined parameters of the algorithm optimized the number of targeted SNP islands from the exhaustive options available throughout the human genome. Under SNP island search parameters, suitable target regions were located, consisting of a high-variance segment of sequence with a defined maximal length, flanked by low-variance (conserved) sequence of defined minimal length.

The algorithm parameters dictated the requirements associated with categorizing a segment as being low-variance or high-variance. The maximum length of a high-variance segment, the minimum number of SNPs that a high-variance segment was required to contain, and the minimum length of a low-variance segment were all tunable thresholds set within the algorithm. TIA maintained a state-based model as it scanned through the genome, with each scanned SNP potentially affecting the state of the model. During genome scanning, there were three active states in which the model existed as 1) establishing low-variance segment I, 2) establishing high-variance segment, and 3) establishing low-variance segment II.

TIA began by scanning the SNP contents of the *1000 Genomes Project* Database in a SNP-by-SNP manner along the human reference genome to find relevant targets. The algorithm first located a span of at least the specified sequence length where no relevant SNPs existed, establishing the Low-Variance Segment I state. Once this segment was established, TIA began to incrementally count the number of impactful SNPs as it moved along the genome sequence until either the maximal user-defined target window length was reached or a user-defined number of impactful SNPs was reached, establishing the High-Variance Segment state. For this study, impactful SNP targets were defined as those with a reported global frequency within the threshold of 30% to 70% variance. If the maximal target window length was reached prior to counting the defined number of impactful SNPs, TIA regressed to the establishing Low-Variance Segment I state. If the impactful SNP count was reached within the maximal window length, TIA advanced to the establishing Low-Variance Segment II state. In this last state, TIA again looked for a span of sufficient base length where no relevant SNPs existed; however, this second span was required to be located before the overall target window exceeded the maximum allowed length. If a low-variance segment was not found within the specified overall region length threshold, TIA regressed to the establishing Low-Variance Segment I state. If a segment of sufficient base length was found, the location of the target window was recorded, and TIA reset to the beginning state, establishing Low-Variance Segment I.

TIA accepts user-defined variables for tailored genome searches, including the minimum length for a low-variance segment, the maximum length of a high-variance segment (identity-linked target window), the frequency range of relevant SNPs based on global SNP frequency, and the minimum number of impactful SNPs to establish a high-variance segment. The TIA process was performed for SNP islands containing a minimum of five, four, and three identity-linked SNPs, occurring within a high-variance sequence window of less than 400 bp. Low-variance flanking regions were limited to a minimum length of 150 bp.

### SNP island quality filtering

SNP island targets containing repeat stretches or patterns of the same nucleotide composition, that potentially interrupt Illumina DNA sequencing chemistries [17, 18], were separated from the pool of viable targets. This filtering was applied as a post-processing step in the TIA Python script, where each discovered target was subjected to predicate logic that determined its suitability. In the algorithm, all targets were scanned along their sequence length in a 20-base pair sliding window, advancing down the sequence in single-base increments. Within each window, if there existed a cluster of a single nucleotide containing a total of ten bases represented within the window, the target was rejected. A single-nucleotide cluster was defined within the algorithm as a string of four or more of the same nucleotide. Repetitions of short nucleotide patterns, defined as being a unique segment of bases whose length was between two and seven bases, were identified within the SNP islands by comparing the target sequence to itself under that range of base offsets. Any block of sequence with repeating bases of the defined length discovered by this comparison were identified as containing a potential repeating pattern. Each of these segments were examined for repetitious patterns, and if a base pattern was found to occur more than five times consecutively along the sequence, the entire target was rejected.

### Primer design feasibility filtering

Primers for targeted amplification of surviving SNP islands were designed to fall within the 150-bp flanking regions of the targets. A separate Python module was developed and used to evaluate the flanking regions of the SNP islands, determining conserved segments within the flanking regions and retaining only those SNP islands meeting the conservation stringency criteria for both flanking regions. Though this algorithmic filter is conceptually a single operation, it was performed in a four-step process. The algorithm located all unique subsequences within the flanking regions of all previously identified target regions, as compared to the entire genome. These subsequences had a minimum sequence length of 15 bp. All SNP islands, lacking unique subsequences in one of the flanking regions, were discarded. The identified unique subsequences from the remaining SNP islands were compared to the human genome reference sequence (version GRCh37.p13; GenBank assembly accession GCA_000001405.14) for similarity of sequence using the BLASTn tool of the *National Center for Biotechnology Information*. SNP islands were discarded from the viable collection if they did not contain flanking region subsequences larger than 25 unique base pairs with no similarity to other regions within the reference genome. Any SNP island with at least one unique sequence for each flanking region was retained.

### Validation of SNP islands for targeted amplification within the human genome

#### Human genomic DNA preparation

Human genomic DNA (gDNA) was collected as buccal swabs from 15 contributors using SecurSwab DUO-V collectors (Bode Technologies). DNA was extracted from the buccal swabs, using the QIAamp DNA Mini Kit (Qiagen) according to the application notes for DNA purification from buccal swabs (version 05/2016). The absolute concentration of recovered human gDNA was quantitated by droplet digital polymerase chain reaction (ddPCR). Reactions were constructed using 10 μL of ddPCR Supermix for Probes containing no dUTP (Bio-Rad), 2 U of *Hind*III (New England Biolabs), and 6 μL of UltraPure DNase/RNase-free water (Thermo Fisher Scientific). 1 μL of each target-specific probe was used within the dual-target reaction. VIC fluorophore-labeled TaqMan copy number reference probe for telomerase reverse transcriptase gene (TERT) (Thermo Fisher Scientific) was used as a single-target autosomal marker, and FAM fluorophore-labeled TaqMan copy number probe for sex-determining region Y gene (SRY) (Thermo Fisher Scientific) was used as a single-target Y-chromosome sex-linked marker. Buccal swab DNA samples (4 μL) were evaluated within their respective reactions in an empirically determined dilution range of 10^− 2^, 10^− 3^, and 10^− 4^.

Droplets were generated using Automated Droplet Generation Oil for Probes (Bio-Rad) in an Automated Droplet Generator (Bio-Rad). DNA within droplet reactions was amplified using a C1000 Touch Thermal Cycler (Bio-Rad). Droplets were evaluated for the presence of target-amplified fluorescence using a QX200 Droplet Reader with excitation wavelengths at 494 nm (FAM) and 538 nm (VIC). Data acquisition and analyses were performed on two fluorescence channels (518 nm and 554 nm) using the QuantaSoft software (Bio-Rad), where concentrations (copies/μL) of the TERT and SRY targets were calculated for the undiluted DNA samples. Reactions were performed in triplicate, and measured DNA concentrations were converted from copies/μL to pg/μL using the conversion estimate of 3.3 pg/haploid copy of the human genome [19].

### Targeted validation and multiplex amplification of SNP island primers

Primer sets were computationally designed for the unique sequences of the low-variance SNP island flanking regions using the SeqBuilder module of the DNASTAR software suite (version 11.2.1.25). During the searches, primer length range was set to between 25 and 45 bp with a target annealing temperature of 70 °C. Identified primers were compared to the NCBI GenBank database human reference genome (version GRCh37.p13) using BLASTn.

Multiplexed targeted amplification was used to amplify SNP island profiles from the genomic DNA (gDNA) of 15 contributing individuals. Contributor gDNA for each individual, ranging from 25 ng to 300 pg of starting DNA mass, was amplified in multiplexed reactions. 27 primer sets from those identified by TIA were selected for the evaluations. Each reaction (50 μL total volume) was composed of 25 ng of contributor DNA, 1 U of Phusion High-Fidelity DNA polymerase, 400 μM of each dNTP, and 0.5 μM of both forward and reverse primers for 27 SNP island targets. Thermal cycle conditions were 98 °C for 3 min, followed by 40 cycles of 98 °C for 10 s, 70 °C for 3 min, and 72 °C for 1 min, with a final extension at 72 °C for 5 min. After amplification, the multiplex reactions were cleaned using AMPure XP reagent (Beckman Coulter), using a 1.8× bead to total volume ratio and eluting in a volume of 50 μL of UltraPure DNase/RNase-free water. The primer specificities and amplicon sizes were evaluated using a DNA 1000 assay chip (Agilent Technologies) on a BioAnalyzer 2100 (Agilent Technologies).

### Illumina MiSeq amplicon sequencing

Illumina paired-end sequencing libraries were prepared using the Accel-NGS 2S DNA Library kit for Illumina Platforms (Swift Biosciences), following manufacturer protocol version 2.0. Each individual DNA pool was given a unique multiplex identifying adapter (MID). Quality and concentration evaluations for amplicon pools, pre- and post-adapter addition, were visualized using a DNA 1000 Assay chip on a BioAnalyzer 2100. Library quality was assessed using the ddPCR Library Quantification Kit for Illumina TruSeq (Bio-Rad), according to manufacturer specifications, on the ddPCR platform. Prepared libraries were sequenced on an Illumina MiSeq platform at the Institute for Genome Sciences Genomics Resource Center at the University of Maryland School of Medicine. The libraries were sequenced in a paired-end manner using the MiSeq Reagent Kit v3 (Illumina, Inc.), generating paired-end 300-bp read lengths.

### Computational post-processing of DNA sequence reads

Paired sequence read files generated from Illumina sequencing were organized and binned into paired, sample-specific files according to MID sequences. The paired files were evaluated and trimmed based on sequence quality using Trimmomatic [20] in paired-end mode. Low quality sequences, sequencing artifacts, sequencing adapters, and MIDs were removed from the reads. The Trimmomatic filter settings included seed mismatches tolerance set to 0, a palindrome clip threshold set to 40, a simple clip threshold set to 15 bp, minimal adapter length set to 8 bp, Phred values set to a minimum of 20 for leading and trailing bases, a sliding window length was set to 4 bp with a minimum Phred score set to 25, and the minimum length of a read was set to 70 bp.

Quality-trimmed reads for each sample were aligned in a paired-end sequence alignment to the human reference genome (version GRCh37.p13) using the Burrows-Wheeler Aligner Maximal Exact Matches (BWA-MEM) program [21]. The resulting sequence/alignment map (SAM) files were revised directly with a Python script to remove any sequences extraneous to the SNP island targets, resulting in a new SAM file with the remaining sequences. The SAM file was converted to a sorted binary-sequence/alignment map (BAM) file using the import command of the SAMtools suite version 0.1.19 [22]. The BAM files were sorted and indexed with the sort and index commands of the SAMtools suite, respectively.

SNP calls were performed using the sorted and indexed BAM files in the Genome Analysis Tool Kit Haplotype Caller [23] and output as allele files in the variant call file format. The nucleotide base call and depth of coverage (DOC) at each SNP position were calculated using the mpileup tool in the SAMtools suite, creating target pileup histograms.

### SNP profile analysis

The DOC at each sequenced base position was used to determine statistical confidence intervals for each haplotype call for each sample. The independence of the haplotype call and the DOC allowed a binomial distribution with a mean of π, where π is the proportion of reference calls, and a variance of *n*π(1-π) for the population distribution. The sample proportion (*p*) of calls that were returned as reference was calculated as

$$ p=x/n $$

, where *x* is the number of times a call is classified as reference and *n* is the number of times that a given SNP is sequenced or DOC. To determine if the sample size was large enough to use the normal distribution to calculate a statistical confidence interval for the sample proportion, the criteria

$$ np\left(1-p\right)\ge 10 $$

was used. If this condition was met, then the use of the normal distribution was determined to be appropriate; otherwise, a confidence interval was not calculated. The confidence interval for the population proportion (π) for all sample proportions that met the sample size criterion was calculated using

$$ p\pm {Z}_{\raisebox{1ex}{$\propto $}\!\left/ \!\raisebox{-1ex}{$2$}\right.}{\sigma}_p $$

, where the standard deviation of *p* is

$$ {\sigma}_p=\sqrt{\frac{p\left(1-p\right)}{n}} $$

and for a 95% confidence level α = 0.5 and *Z* = 1.96. *Z* is a standardized normal random variable with a mean of 0 and a standard deviation of 1. *Z* measures the number of standard deviations that an observation is from the mean. For a 95% confidence level (CL), α = 1-CL = 0.5. The corresponding *Z* value is 1.96, which is found using a standard normal probability distribution table [24].

The distribution of haplotype calls for SNP classifications within a given profile was visualized by scatterplot of the sample *p* and the confidence limits (margin of error) for each SNP location using the ggplot2 package [25] and by marginal histogram using the ggExtra package [26] of the *R* statistical software [27]. Genomic SNP position profiles with confidence intervals that included or were less than *p* ≤ 0.1 were classified as homozygous variant, that included or were greater than *p* ≥ 0.9 were classified as homozygous reference, and that included 0.4 ≤ *p* ≤ 0.6 were classified as heterozygous.

The relation of the SNP profiles between the individuals was visualized based on the zygosity determination for each SNP location. SNP profile calls were assigned values of 1 for the homozygous reference zygosity, 2 for the heterozygous zygosity, or 3 for the homozygous variant zygosity. A total of 116 SNP locations within 28 SNP islands for each profile were evaluated. The relationship between the zygosity profiles of identity-relevant SNP locations for fifteen individual genomic samples were evaluated by the percent similarity, calculated as the number of matched SNP calls divided by the total number of calls between the individual pair.

A haplotype heatmap and relatedness dendrogram were generated using the heatmap.2 function of the gplots package [28] in the *R* statistical software. The zygosity of each evaluated SNP location for each profile was represented as a green band (homozygous reference), a yellow band (heterozygous), or a red band (homozygous variant). The Euclidean distance of the relatedness of the SNP zygosity profiles and the complete agglomeration method for clustering were used to construct an agglomerative hierarchical clustering dendrogram.

## Results

### Algorithmic identification of identity-linked SNP islands

Using the parameters for identity-linked SNP island discovery, the computational algorithms developed within this study located 54 qualifying SNP islands for use with the Illumina MPS platform chemistry. In a stepwise manner, the target identification filter (79,154 targets remaining), sequence repetition rejection filter (29,465 targets remaining), and the primer design feasibility filter (54 targets remaining) narrowed the scope of regions for targeted SNP island amplification within the genome (Table 1). Of these, twelve 5-SNP islands, eight 4-SNP islands, and thirty-four 3-SNP islands were identified. A total of 309 identity-relevant SNPs, including those identified within the *1000 Genomes Project Database* and those identified in this study, were located within the SNP islands (Additional file 1: Table S1). Of the identity-relevant SNPs characterized, 166 were previously described in the database and had reported global allele frequencies of 30–70% as defined by TIA. 51 SNPs had a described SNP location but fell outside the TIA-defined global allele frequency, while 18 SNPs had a described SNP location but an unknown frequency. Within this study, 74 SNPs were characterized as SNPs that were not previously defined within the *1000 Genomes Project Database*.

**Table 1:** Identity-Linked SNP Island Identification within the Human Genome using Computational Algorithms

	Target Identification Filter			Sequence Repetition Rejection Filter			Primer Design Feasibility Filter		
Chromosome	5-SNP	4-SNP	3-SNP	5-SNP	4-SNP	3-SNP	5-SNP	4-SNP	3-SNP
**1**	311	1231	4557	126	478	1689	3	0	2
**2**	350	1185	4666	131	444	1759	2	0	1
**3**	288	1085	3973	116	417	1530	0	0	6
**4**	368	1218	4306	137	463	1699	0	0	4
**5**	256	939	3546	80	328	1314	0	1	0
**6**	289	987	3717	104	374	1414	1	1	3
**7**	255	933	3393	79	326	1244	0	2	0
**8**	203	748	3015	83	297	1229	1	1	2
**9**	167	581	2333	55	192	902	1	0	0
**10**	230	819	2891	76	300	1143	1	0	0
**11**	228	754	2878	96	299	1197	0	0	2
**12**	201	732	2593	62	261	967	1	0	2
**13**	181	608	2179	62	221	797	0	0	2
**14**	120	469	1748	32	168	641	1	0	1
**15**	129	446	1653	44	152	617	0	0	0
**16**	140	416	1630	41	144	589	0	1	1
**17**	123	446	1654	37	151	566	0	1	2
**18**	123	444	1597	54	164	587	0	0	1
**19**	126	411	1399	36	99	362	0	0	0
**20**	108	377	1372	38	139	521	0	0	3
**21**	93	302	993	28	111	370	0	0	2
**22**	60	199	751	23	68	275	1	0	0
**X**	119	485	2027	41	154	722	0	1	0
**Y**	0	0	0	0	0	0	0	0	0
**TOTAL**	**4468**	**15,815**	**58,871**	**1581**	**5750**	**22,134**	**12**	**8**	**34**
**Remaining Target Islands**	**79,154**			**29,465**			**54**		

The human reference genome (version GRCh37.p13) was computationally filtered for unique, identity-linked SNP islands, containing a minimum of 5, 4, or 3 identity-relevant SNPs. SNP islands amenable to analysis by Illumina sequencing chemistries and to targeted amplification from the genome were located using a target identification filter, a sequence repetition rejection filter, and a primer design feasibility filter

### Genomic DNA sample preparations and SNP island validations

Computational prediction produced 90 unique primer sets, designed for the 54 SNP island targets identified in the filtering algorithms. Primer pairs amplifying more than one genomic region or producing unexpected amplicon sizes from genomic DNA, using described conditions in singleplex PCRs, were eliminated as non-targeted for the desired SNP island region. As a result, 54 targeted primer sets were functionally accepted (Additional file 1: Table S1).

### MiSeq run summary statistics

**Fig. 2:**
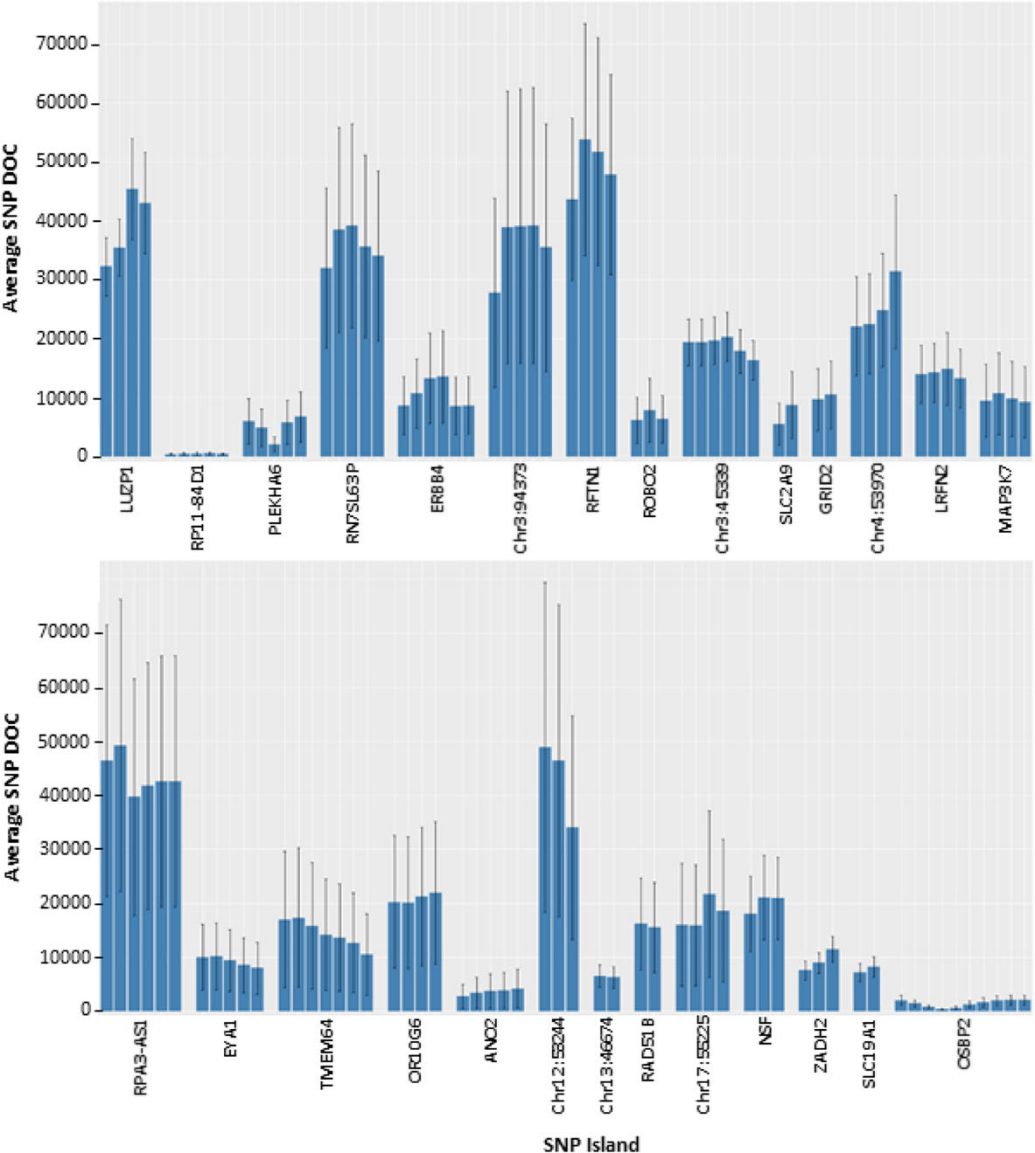
SNP island amplification and sequencing efficiency. The depth of coverage (DOC) for each identity-linked SNP location within 27 SNP islands was averaged across all contributing individuals. Error bars represent variance within a 95% confidence interval

The sequence evaluation of SNP island amplicons using the Illumina MiSeq DNA sequencing platform identified 12,379,802 sequence reads (3714 megabases) at an average trimmed read length of 240 bp. Figure 2 illustrates the average DOC for each identity-linked SNP location for 27 SNP island targets. Variability of DOC observed within select island targets (Fig. 2) was observed across the 15 contributing genomes, indicating efficiencies of island amplifications.

### Zygosity profile determination

**Fig. 3:**
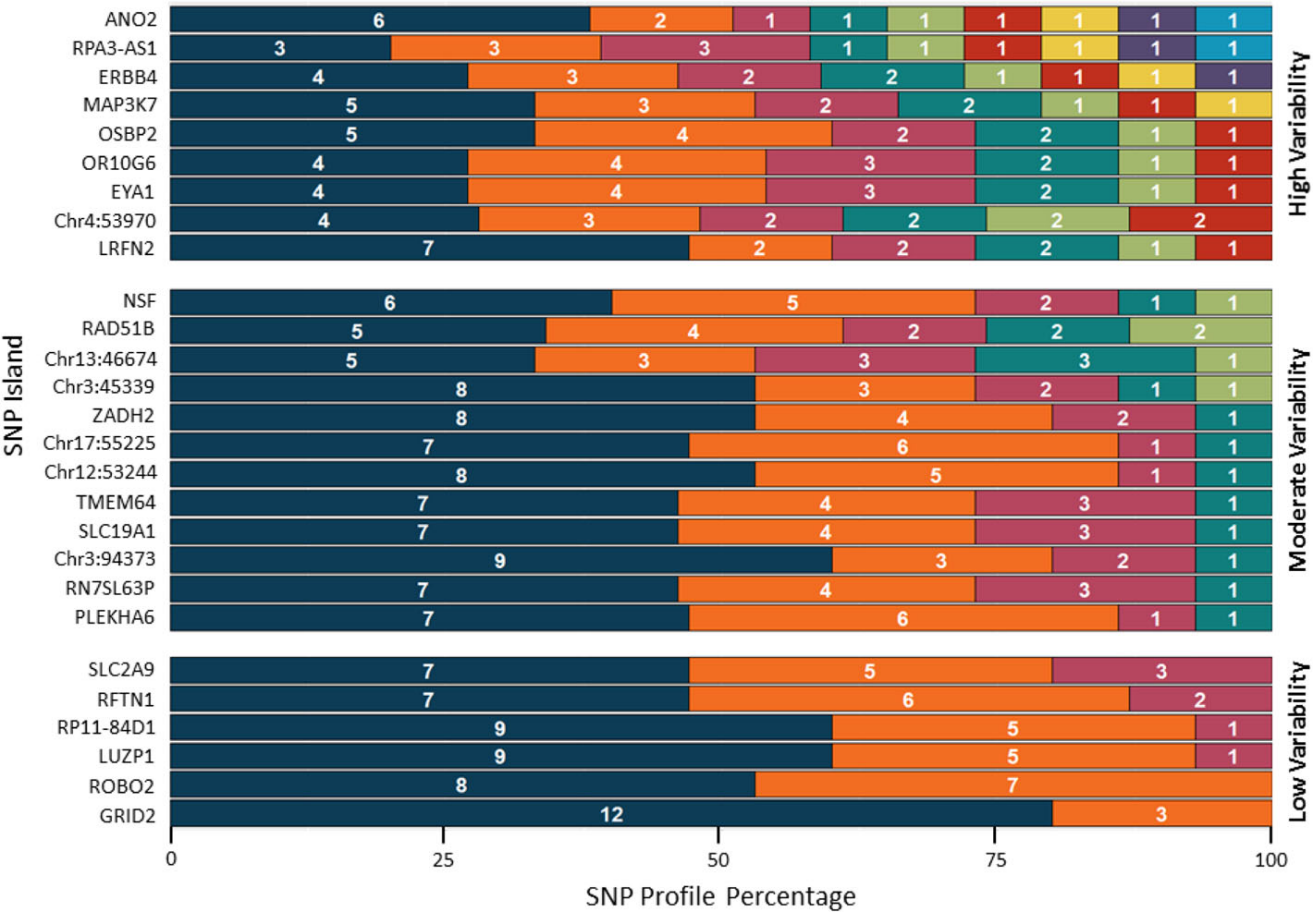
Profile variance within SNP islands across individuals. The SNP profile for each individual at the SNP island level was evaluated to determine the variability of SNP profiles within SNP islands. SNP islands are grouped as containing high variability (≥6 species), moderate variability (4–5 species), and low variability (≤3 species) according to the numbers of haplotype island species observed across all individuals. The numbers within the bars represent the absolute number of observed SNP island species

**Fig. 4:**
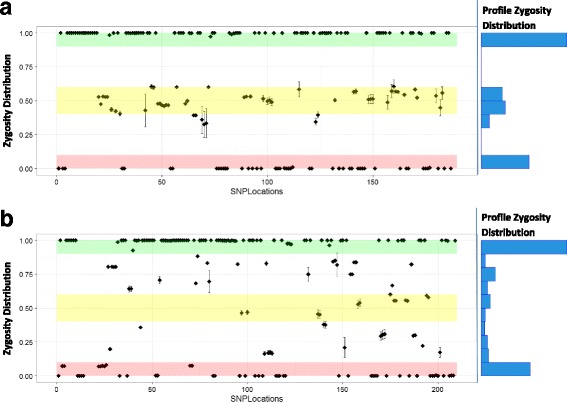
Zygosity comparison between a single-contributor and a multiple-contributor sample. Depth of coverage for SNP calls was used to determine the proportion (*p*) of the zygosity of the given SNPs within the profiles. Visualization of the zygosity proportions across the entire SNP profile as they map to defined zygosity regions allows the differentiation of single- and multiple-contributor samples. The plotted proportions of the single-contributor sample (**a**) constrain to the defined regions of homozygous reference (0.9–1.0 *p*, green), heterozygous (0.4–0.6 *p*, yellow), or homozygous variant (0–0.1 *p*, red) for the evaluated SNP locations. The plotted proportions of the multiple-contributor sample (**b**) are distributed widely between the homozygous extremes and are not constrained to the defined zygosity regions

Using the DOC for the same 27 SNP locations, the variation in zygosity pattern within each SNP island was calculated across all individuals (Fig. 3), indicating islands with low, moderate, and high variabilities. In addition, the probability of the sample zygosity for each SNP location was determined and plotted in a scatterplot for each sample. Figure 4 illustrates the variation that is observable between a single-contributor and mixed-contributor (1:1 ratio) sample. The zygosity probability for each SNP location in the single-contributor sample of Fig. 4a falls within the defined regions considered appropriate for zygosity calls of homozygous reference (0.9–1.0 *p*, green), heterozygous (0.4–0.6 *p*, yellow), and homozygous variant (0–0.1 *p*, red). Figure 4b illustrates the variability in DOC and, as a result, the probability of zygosity call for each SNP location with many of the SNP calls falling outside the defined regions of expected zygosity. In comparison of the profile zygosity probability distributions between the two samples, the single-contributor sample contains probabilities that create three defined zygosity regions within the distribution, while the multiple-contributor sample contains representation across the scope of observable zygosity probability.

### Profile comparative analysis

**Fig. 5:**
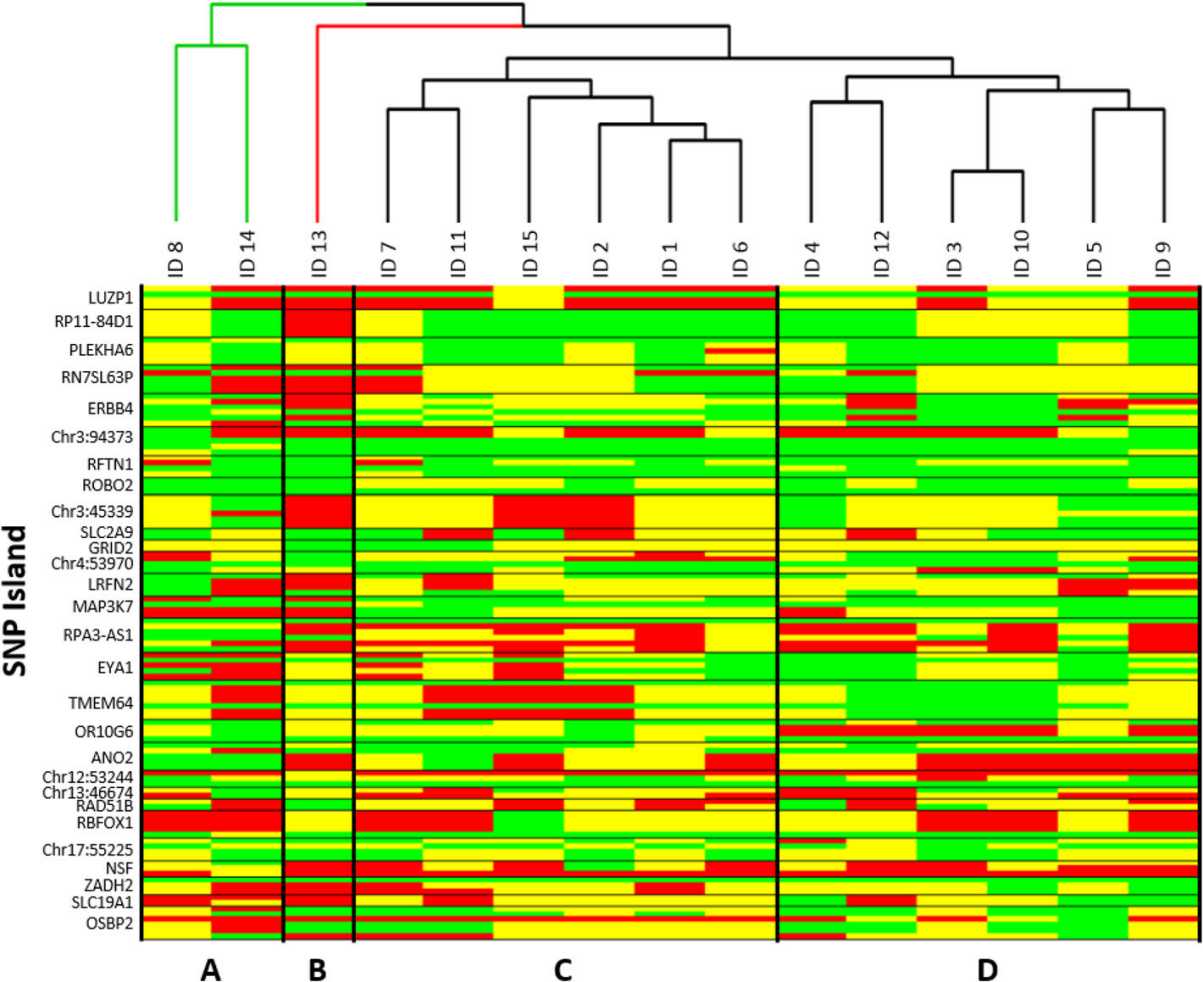
Zygosity Profile Comparison at Identity-Linked Loci. Zygosity comparisons, using the identity-linked SNP island panel compiled with algorithms of this study, differentiated the genomes of fifteen individuals. A total of 116 SNP locations within 28 SNP islands were evaluated in the heatmap profile comparison with homozygous reference zygosity represented as a green band, heterozygous zygosity represented as a yellow band, and homozygous variant zygosity represented as a red band. The accompanying agglomerative hierarchical clustering dendrogram represents the Euclidean distance of profile relatedness between the individual SNP zygosity profiles, resulting in three branches denoted in the dendrogram as green, red, and black lines. Three clusters (**a**, **c**, and **d**) and one outlying singleton (**b**) were identified

Fifteen single-contributor SNP profiles (116 identity-relevant SNP locations) generated from the identity-linked SNP island targets were evaluated for similarity (Table 2) and SNP profile relatedness (Fig. 5). Each individual was uniquely identified from all other individuals to varying degrees in the comparisons. The zygosity similarity comparison, represented in Table 2, showed the highest similarity between two profiles within the total fifteen profiles to be 82% similar, sharing 95 of 116 SNP location calls. The lowest similarity between two profiles was 35% similar, sharing only 41 of 116 SNP location calls. For the pool of SNP profiles, the typical profile zygosity similarities were between 40% and 60% similarity. The heatmap of the SNP profile relatedness of fifteen individuals (Fig. 5) illustrates the similarities and differences for each profile at 27 SNP locations evaluated. The results indicate that each individual has a unique SNP profile as compared to others in the pool. The agglomerative hierarchical clustering dendrogram of SNP profile relatedness is represented by three branches containing three clusters and one outlying singleton.

**Table 2:** Similarity matrix of SNP data for evaluated individuals

	ID1	ID2	ID3	ID4	ID5	ID6	ID7	ID8	ID9	ID10	ID11	ID12	ID13	ID14	ID15
ID1	―	72	62	69	47	78	61	59	70	59	62	72	52	46	63
ID2	0.62	―	66	60	51	71	59	46	56	57	60	54	55	51	69
ID3	0.53	0.57	―	48	54	70	60	56	61	95	62	58	50	51	58
ID4	0.59	0.52	0.41	―	70	68	51	59	60	53	55	70	55	49	50
ID5	0.41	0.44	0.47	0.60	―	64	58	61	63	59	52	56	61	41	58
ID6	0.67	0.61	0.60	0.59	0.55	―	66	64	62	56	57	66	52	45	60
ID7	0.53	0.51	0.52	0.44	0.50	0.57	―	61	50	62	66	50	64	48	49
ID8	0.51	0.40	0.48	0.51	0.53	0.55	0.53	―	54	52	54	47	43	43	44
ID9	0.60	0.48	0.53	0.52	0.54	0.53	0.43	0.47	―	61	67	52	47	48	62
ID10	0.51	0.49	0.82	0.46	0.51	0.48	0.53	0.45	0.53	―	63	67	53	47	59
ID11	0.53	0.52	0.53	0.47	0.45	0.49	0.57	0.47	0.58	0.54	―	63	51	62	61
ID12	0.62	0.47	0.50	0.60	0.48	0.57	0.43	0.41	0.45	0.58	0.54	―	53	42	66
ID13	0.45	0.47	0.43	0.47	0.53	0.45	0.55	0.37	0.41	0.46	0.44	0.46	―	48	48
ID14	0.40	0.44	0.44	0.42	0.35	0.39	0.41	0.37	0.41	0.41	0.53	0.36	0.41	―	50
ID15	0.54	0.59	0.50	0.43	0.50	0.52	0.42	0.38	0.53	0.51	0.53	0.57	0.41	0.43	―

Haplotype similarities of samples ID1-ID15 for each SNP location are represented beneath the diagonal, while the number of SNP haplotypes shared between pairs of samples are above the diagonal

## Discussion

Driven by natural selection, the genetic diversity of the modern human population spans the globe and results in unique inheritable markers of distinction within the population [29]. These genetic variations result in over 84 million known single nucleotide polymorphisms (SNPs), defining the worldwide human populations [13]. These unique patterns of SNP inheritance, governed by factors of genetic heredity, physical isolation, and environmental influences, account for the diverse and unique SNP patterns displayed within the human population and among individuals.

SNPs are widely reported as having utility for determining identity, ancestry, phenotype, and disease states [7, 30, 31]. As the forensics and medical communities pivot to leverage the information contained within these genomic markers, databases with allele frequency data, representing a larger portion of the human population, are required to determine the frequency of the given genetic variation across the global human population. Databases like the *1000 Genomes Project SNP Database* [13], the *Database of Single Nucleotide Polymorphisms* (dbSNP) [32], the *International HapMap Project* [1, 14], and the *Allele Frequency Database* (ALFRED) [33] continue to develop and expand, describing the SNP variations of global populations with greater fidelity. These databases are useful for predicting the application of newly-developed SNP panels across a broader subset of the global human population range [7]. The allele frequencies of known and characterized SNPs provide a basis for determining genomic regions with utility for identifying and describing characteristics of individuals, using markers within their genomes. Tailored SNP panels for answering targeted questions can be developed from these data.

In this study, a target identification algorithm (TIA; Fig. 1) was designed to locate discrete regions within the human genome based on global allele frequency data found in the *1000 Genomes Project Database*. According to tunable parameters, the Python algorithms focused on SNP island target selections that not only contain identity-relevant SNPs but also are amenable to analysis using the Illumina MPS platform. The filtering algorithms located identity-linked SNP islands in the human genome, of which 54 islands continued forward within the study after trial validation screening. In application of the 27 islands, the average DOC for evaluated identity-linked SNP locations indicated variability in the amplification and sequencing efficiencies across the SNP islands (Fig. 2). While most of the regions were amplified and sequenced at a high average DOC (> 10,000 reads) for diluted starting genomic samples, the SNP islands of RP11-84D1, ANO2, Chr13:46674, and OSBP2 were consistently analyzed at average DOCs well below 10,000 reads. Although base calls were made at the respective SNP locations, the non-uniform amplifications of these SNP locations indicate variability in the specificity of the target primer sets or sequencing error due to sequence base content. As a result, the affected SNP islands are indicated as low performers and may require modification or exclusion to achieve a robust multiplex reaction.

Though the Illumina sequencing platform presents limitations with its chemistry and short-read assembly [34, 35], the platform was selected for this study for its simple workflow and scalability. Paired-end Illumina DNA sequencing was used to allow detection of rearrangements and sequence variances such as insertions, deletions, and inversions [36]. The open availability of bioinformatics tools for computational post-processing of DNA sequence reads provided a systematic process for contiguous sequence fragment reconstruction and SNP identification within the individual reads. The SNP island discovery process was focused by tunable parameters, such as SNP island sequence length and numbers of variants it contains as well as their conserved flanking region characteristics. These constraints allow TIA to select sequences that would conform to the Illumina sequencing chemistry. In accomplishing this, TIA defined a high-variance segment of sequence, or SNP island, as a stretch of bases not exceeding 400 bp. Each high-variance segment contained at least the minimum number of impactful SNPs, where an impactful SNP was defined as one with reported global allele frequency within a threshold of 30% to 70% variance. Further, the algorithm defined flanking low-variance sequences as those containing at least 150 bp of conserved sequence with SNP frequencies of ≤0.5% or ≥ 99.5%. Manipulation of the filter parameters permits the modification of SNP island target regions and their conserved flanking regions, allowing the user to quickly design SNP island targets that are tailored to the application.

TIA can be expanded beyond identity-linked SNPs to include ancestry or phenotype. For example, SNP island discovery using TIA can be designed using population-specific characteristics such as ancestry or ethnicity for the development of a targeted SNP panel, selective for a given population type. It has been noted that one of the most important criteria of an informative SNP is its compatibility with sequencing chemistries [12]. To address this, the algorithmic parameters in the TIA genome filters can be modified to accommodate other sequencing chemistries like those of Pacific Biosciences and Ion Torrent, requiring sequence specifications that differ from those of the Illumina platform (data not shown).

Each SNP island contained a minimum of 3, 4, or 5 identity-relevant SNPs as determined by global frequency calculations represented within the *1000 Genomes Project Database*. As the SNP profiles of individual contributors were defined for the SNP island regions, 143 identity-relevant SNPs were recognized beyond those selected by the algorithms. In some instances, these additional SNPs did not meet the thresholds defined by the algorithmic filters of 30%–70% global frequency, and in other instances, the SNPs were not defined within the *1000 Genomes Project Database*, indicating the detection of previously undefined SNPs (Additional file 1: Table S1). These results highlight the known limitations of databases like the *1000 Genomes Project Database* for under-representing the SNP allele frequencies of the global human population [13]. Expansion of the *1000 Genomes Project Database* and other similar databases have been noted as a means to provide a greater resolution to the global human genome variance [1, 13]. As these types of databases continue to expand, the numbers of identified SNPs and their relative global population frequencies will gain in fidelity.

Evaluation of the SNPs from the suite of 27 islands selected to differentiate fifteen individuals provided insight into the degree of individual discerning power within each SNP island (Fig. 3). As anticipated, there were varying efficiencies between regions chosen for target islands, observing variance at low, moderate, and high occurrences. Of the islands evaluated against contributor gDNA, island targets *ERBB4*, *MAP3K7*, *RPA3-AS1*, and *ANO2* contain a higher profile variance than other SNP islands, indicating a higher recombination frequency within the sequences of those islands. The higher variance within these islands across contributors makes them more powerful for discerning individual identity.

Increasing the number of identity-relevant SNPs contained within each sequenced target provided more identity-discerning information for each SNP island. In the evaluation of 27 identity-linked SNP islands across the genomes of fifteen individuals, the SNP variation, contained within a subset of the total number of SNP islands located by TIA, effectively differentiated the identities of all individuals assessed (Table 2 and Fig. 5). Evaluating the SNP location DOC statistics from the MPS run also provided a means to differentiate between single-contributor and multiple-contributor samples (Fig. 4). The zygosity proportion distribution for the single-contributor sample conformed tightly to the three expected zygosity determinant regions of homozygous reference (0.9–1.0 *p*), heterozygous (0.4–0.6 *p*), and homozygous variant (0–0.1 *p*). The variance from the three expected zygosity determinant regions observed for the multiple-contributor sample indicated a mixed sample with alternate DOCs and the resulting distribution proportions overlaying each other.

In comparison of 15 single-contributor samples, the similarity of the resulting evaluated SNP profiles produces a typical similarity score between 40% and 60% (Table 2). This observed range falls well within the global SNP frequency range of 30% to 70% targeted by the TIA, indicating that the algorithms effectively selected identity-relevant SNP targets. The heatmap representation and accompanying dendrogram of the individual SNP profiles (Fig. 5) provides a means to visualize the individual SNP location differentiation between the SNP profiles while grouping the profiles by overall relatedness. While distinct groups were observed, each individual profile was easily differentiated from all other profiles. In an additional application of the SNP islands for the differentiation of related individuals (data not shown), the identity-linked SNPs uniquely identified individuals of sibling and parent-child pairs.

The algorithmic narrowing of the scope of genomic regions provided SNP island options conducive to analysis by targeted amplification. In addition, the algorithms allowed the efficient evaluation of the greater human genome for regions that were amenable to uniquely identifying individual contributors within the Illumina MPS workflow. These algorithms were developed in a manner that makes them tunable for determining the desired genomic features. The operator can change the maximum length of the SNP island, the minimum length of the low-variance primer regions, the number, frequency, and type of repeat sequences allowed within the SNP island, and the minimum number of known SNPs having a defined global or population-specific frequency range. By tuning the algorithms to desired target goals, the resulting SNP panel can be applied to discovery of identity-, ancestry-, or phenotype-linked information. In addition, the target islands can be modified to accommodate the requirements and advantages of other sequencing platform chemistries.

Systems and methods like ForenSeq [8] for the Illumina MPS platform and Ion AmpliSeq HID SNP panel [12] for human identification for the Ion Personal Genome Machine MPS platform are under evaluation for application in genetic profiling within forensic casework. While these methods provide a standardized, pre-defined methodology for determining a given individual profile, the systems are limited in their ability to tailor the assays for differentiations at higher resolution and with regard to multiple contributor samples . TIA provides a tunable system that can be customized by the user.

## Conclusions

TIA provides a genome analysis tool for the selection and development of target regions within the genome, leveraging SNP locations and population frequency information in SNP databases. The target regions discovered by TIA are tunable for the target sequence length and quality, conserved flanking sequence length, allowable variability within the conserved flanking sequence, the global population frequency of the target region SNPs, and the minimum and maximum number of qualifying SNPs contained within the target regions. The target regions identified by TIA are amenable to evaluation by targeted resequencing using MPS platforms. The advantages of TIA are that it allows the user to develop tailored SNP islands, according to SNP allele frequency and population information. As a result, the algorithms provide flexibility to rapidly identify and validate new informative SNP panels as global population SNP databases mature and gain in fidelity and as new MPS technologies come online.

## Additional file

Additional file 1: **Table S1**. Identity-Linked SNP and SNP Island Genomic Locations. Reported global allele frequencies are those represented within the *1000 Genomes Project Database*. For SNP loci labeled NSV, no SNP variant was previously reported for that genomic location. Deletion events are represented as a dash (−). (DOCX 56 kb)

## Abbreviations

BAM: Binary-sequence/alignment map
ddPCR: Droplet digital PCR
DNA: Deoxyribonucleic acid
dNTP: Deoxynucleotide triphosphate
DOC: Depth of coverage
FAM: Carboxyfluorescein
gDNA: Genomic DNA
HID: Human identification
MID: Multiplex identifying adapter
MPS: Massively parallel DNA sequencing
NSV: No SNP variant
*p*: Sample proportion
PCR: Polymerase chain reaction
SAM: Sequence/alignment map
SNP: Single nucleotide polymorphism
TIA: Target identification algorithm
