# Supplementary material for: TIA: algorithms for development of identity-linked SNP islands for analysis by massively parallel DNA sequencing
Source: BMC Bioinformatics. 2018 Apr 11;19:126. doi: 10.1186/s12859-018-2133-2 (PMC5896139; doi:10.1186/s12859-018-2133-2)
Supplement: Supplementary file 1 — Table S1. Identity-Linked SNP and SNP Island Genomic Locations. Reported global allele frequencies are those represented within the 1000 Genomes Project Database. For SNP loci labeled NSV, no SNP variant was previously reported for that genomic location. Deletion events are represented as a dash (−). (DOCX 56 kb) [file 12859_2018_2133_MOESM1_ESM.docx]

| **Target Name** | **SNP Island Genomic Range** | **SNP Island Size (bp)** | **Identity-Linked SNP Genomic Location** | **SNP Locus** | **Global Allele Frequency** | **Ref** | **Var** | **Forward Primer** | **Reverse Primer** |
| --- | --- | --- | --- | --- | --- | --- | --- | --- | --- |
| LUZP1i | 1:23419097-23419574 | 477 | 1:23419261 | rs477717 | 30.95% (G) | G | A | TACTTGACTTCCGTTGCCAAGCACGTGTCCCCTCTTGC | TCCTCAGCATAAGCGGGAACGACTCCGGAACAGGGAGTTT |
|  |  |  | 1:23419283 | rs35917050 | 2.24% (A) | G | A |  |  |
|  |  |  | 1:23419374 | rs3765407 | 32.31% (G) | G | T |  |  |
|  |  |  | 1:23419383 | rs477830 | 30.97% (C) | C | T |  |  |
| ERBB4 | 2:212264500-212264885 | 385 | 2:212264533 | NSV |  | G | T | TGCTTTGGAGACCTAGAGAACCAGGGTTTAGCTGTATC | TTTAAGGCAGCGGGGCTGTTGGATGACCGTCCTTTTCTCTTA |
|  |  |  | 2:212264534 | NSV |  | T | G |  |  |
|  |  |  | 2:212264560 | rs3791691 | 28.90% (T) | C | T |  |  |
|  |  |  | 2:212264598 | rs3791692 | 36.96% (G) | G | A |  |  |
|  |  |  | 2:212264642 | rs4673613 | 36.24% (C) | T | C |  |  |
|  |  |  | 2:212264646 | rs77175994 | 3.43% (T) | C | T |  |  |
|  |  |  | 2:212264802 | rs4673614 | 35.92% (T) | A | T |  |  |
|  |  |  | 2:212264814 | NSV |  | G | T |  |  |
|  |  |  | 2:212264818 | rs7585000 | 37.70% (C) | A | C |  |  |
|  |  |  | 2:212264855 | NSV |  | G | A |  |  |
| Chr3:94373 | 3:1094373-1094721 | 348 | 3:1094443 | rs2727993 | 48.34% (A) | A | G | ACTTACAACAACTGGAGTTTGGCTTGTCAAGAGTCGTGCAGA | ACTGTCTGTGCTCAATTACCTGCTCCTACACCATCCTACAC |
|  |  |  | 3:1094516 | rs2727994 | 48.30% (A) | A | G |  |  |
|  |  |  | 3:1094524 | NSV |  | G | T |  |  |
|  |  |  | 3:1094526 | rs113173277 | 1.34% (T) | C | T |  |  |
|  |  |  | 3:1094604 | rs12632405 | 17.91% (G) | T | G |  |  |
| RFTN1 | 3:16397692-16398098 | 406 | 3:16397740 | NSV |  | C | A | ACCTGCCCACACTCGCTTGGCATCTTGGACTGAGCTTTACT | GGGGGGTGAGTTGGATGTCCCCCGGGACACTATTACTCAG |
|  |  |  | 3:16397749 | NSV |  | T | C |  |  |
|  |  |  | 3:16397755 | NSV |  | T | C |  |  |
|  |  |  | 3:16397814 | rs140429135 | 4.09% (A) | G | A |  |  |
|  |  |  | 3:16397878 | rs7634895 | 32.11% (C) | T | C |  |  |
|  |  |  | 3:16397921 | rs75696774 | 2.30% (T) | C | T |  |  |
|  |  |  | 3:16397944 | rs117459672 | 4.09% (T) | C/G | T |  |  |
|  |  |  | 3:16398086 | NSV |  | C | T |  |  |
| ARHGEF3 | 3:57010820-57011276 | 456 | 3:57010995 | rs181361690 | 0.08% (A) | G | A | GGGAGTGGAACTGCGTCAGGCTTGTTAGAGCTCATGTGGTAG | GTCATTTTTCTCGCCCCAGTTTGTGGCTCTTGTGGATTAG |
|  |  |  | 3:57011075 | rs9882789 | 46% (G) | A | G |  |  |
|  |  |  | 3:57011075 | rs368071441 | 0.14% (-) | A | - |  |  |
|  |  |  | 3:57011100 | rs183691789 | 0.02% (G) | A | G |  |  |
|  |  |  | 3:57011234 | rs13080107 | 45.89% (C) | T | C |  |  |
|  |  |  | 3:57011234 | rs386661228 |  | TG | CA |  |  |
|  |  |  | 3:57011235 | rs79544289 | 5.01% (A) | G | A |  |  |
|  |  |  | 3:57011235 | rs386661228 |  | TG | CA |  |  |
| ROBO2 | 3:77720194-77720564 | 370 | 3:77720284 | rs4525838 | 37.2% (A) | C | A | GAACACCCTGTTGTTATAGGGATGATAGGATTACAGAGTAAC | TTGCAGTAGCATTGCAGGGAACTTCCGCAAATGCCCCTGT |
|  |  |  | 3:77720336 | rs2324816 | 37.24% (C) | T | C |  |  |
|  |  |  | 3:77720464 | NSV |  | C | A |  |  |
| Chr3:45339 | 3:145145339-145145679 | 340 | 3:145145414 | NSV |  | G | C | ACTGTGGCTCCACGCTCAGCCCATTGTTGGACCTGGCA | AATGCTGTACCACGCTCTTGCTTGCTAAACTGTGAGAGTC |
|  |  |  | 3:145145476 | rs7430464 | 44.27% | G | A |  |  |
|  |  |  | 3:145145477 | rs7430466 | 44.27% | G | A |  |  |
|  |  |  | 3:145145485 | rs62270108 | 44.27% | G | C |  |  |
|  |  |  | 3:145145521 | rs1564488 | 58.95% | A | C |  |  |
|  |  |  | 3:145145605 | rs3916049 | 44.03% | C | T |  |  |
|  |  |  | 3:145145626 | rs3916050 | 44.05% | A | C |  |  |
| Chr3:45379 | 3:145145379-145145679 | 300 | 3:145145414 | NSV |  | G | C | CTGTGAGCGGCTTCAGCATCGGGCGCTGACGTCCAGATGA | AAATGCTGTACCACGCTCTTGCTTGCTAAACTGTGAGAGTC |
|  |  |  | 3:145145476 | rs7430464 | 44.27% | G | A |  |  |
|  |  |  | 3:145145477 | rs7430466 | 44.27% | G | A |  |  |
|  |  |  | 3:145145485 | rs62270108 | 44.27% | G | C |  |  |
|  |  |  | 3:145145521 | rs1564488 | 58.95% | A | C |  |  |
|  |  |  | 3:145145605 | rs3916049 | 44.03% | C | T |  |  |
|  |  |  | 3:145145626 | rs3916050 | 44.05% | A | C |  |  |
| SLC2A9 | 4:9965249-9965750 | 501 | 4:9965432 | NSV |  | C | T | GAGAGGGCCTCTCAAGAGGCCCCTGCTCGCTGGGTTTTGCA | TTGTAAGAATTCTCGAATGTCTCCCCCTCGATATTCAG |
|  |  |  | 4:9965443 | rs6856127 | 41.69% (C) | T | C |  |  |
|  |  |  | 4:9965612 | NSV |  | G | T |  |  |
|  |  |  | 4:9965633 | rs6840802 | 40.93% (G) | C/A | G |  |  |
|  |  |  | 4:9965673 | NSV |  | G | T |  |  |
| ST3GAL1P1 | 4:68586070-68586580 | 510 | 4:68586197 | rs139445314 | 0.10% (G) | C | G | ATAAGTTCTGCAGAGTACATAGTCCAGTCGTTGTGTGAT | TGATGAGAAGCCGGTGGTGAGCAGTTGTACAAGATCCACAG |
|  |  |  | 4:68586267 | rs17635222 | 16.03% (C) | G | C |  |  |
|  |  |  | 4:68586315 | rs13124793 | 14.00% (A) | G | A |  |  |
|  |  |  | 4:68586335 | rs72643632 | 17.79% (C) | T | C |  |  |
|  |  |  | 4:68586335 | rs35801483 |  | TA | CG |  |  |
|  |  |  | 4:68586336 | rs17635292 | 14.22% (G) | A/C | G |  |  |
|  |  |  | 4:68586336 | rs35801483 |  | TA | CG |  |  |
|  |  |  | 4:68586450 | rs7697056 | 34.88% (A) | C | A |  |  |
|  |  |  | 4:68586509 | rs6552111 | 34.88% (T) | C | T |  |  |
| GRID2 | 4:93309655-93310185 | 530 | 4:93309731 | NSV |  | T | C | TACAGTCTACATAATCATGCCCCACAACGGCTATCTTTAGCA | TTTGGGTTAACCAGTATTAAGGGCCGGTTTCCATTACTG |
|  |  |  | 4:93309909 | rs13122638 | 40.64% (C) | T | C |  |  |
|  |  |  | 4:93309955 | rs13122662 | 40.62% (C) | T | C |  |  |
| Chr4:53970 | 4:184453970-184454377 | 407 | 4:184454079 | rs4440262 | 32.83% (G) | T | G | CTGGCAGATGATCCCCTCAGCCAGGTCTAGGGCTTACCCT | TGCTGCCCTTCAGTACCGACTTTGGAAGCAGTACCGAAGT |
|  |  |  | 4:184454084 | rs4518295 | 38.16% (G) | T | G |  |  |
|  |  |  | 4:184454112 | NSV |  | G | T |  |  |
|  |  |  | 4:184454164 | NSV |  | T | A |  |  |
|  |  |  | 4:184454194 | rs7670109 | 46.03% (A) | G | A |  |  |
| MAP3K7 | 6:91318430-91318859 | 429 | 6:91318461 | rs564545140 | 0.02% (T) | C | T | TTGGTGGAAGCTGCGAGTGAGGGGCCTCTCTCGGTGTTTCC | TCAACAATTGTTACCTTAACAAAACCGTTCTAGCCACTTT |
|  |  |  | 6:91318479 | rs9294463 | 38.72% (G) | A | G |  |  |
|  |  |  | 6:91318706 | rs190232649 | 0.26% (T) | C | T |  |  |
|  |  |  | 6:91318722 | rs401147 | 40.46% (T) | T | C |  |  |
|  |  |  | 6:91318768 | rs398026 | 40.46% (A) | A | G |  |  |
| TNKS | 8:9571206-9571735 | 529 | 8:9571364 | rs7462070 | 44.09% (C) | C | T | TGCTATCAGTAGATATCAAATGCCGTAGCAACTCCTATTTAC | GCATGCCTGTCTAGGTTATATATGGCGTACATTTAGTTTA |
|  |  |  | 8:9571399 | rs113085501 | 0.30% (C) | T | C |  |  |
|  |  |  | 8:9571445 | rs7459728 | 30.89% (T) | T | C |  |  |
|  |  |  | 8:9571506 | rs28415429 | 1.98% (G) | C | G |  |  |
|  |  |  | 8:9571621 | rs7461939 | 44.07% (G) | G | A |  |  |
| EYA1 | 8:72111431-72111846 | 415 | 8:72111599 | rs10103397 | 47.66% (G) | A | G | GGCAGAGGTCAAGACTGACAGCAACTGCGCATCACCAGGCGG | CAACTCAGTCCAGTATTCTTAGGGGAGGATTGAGTTTGAAT |
|  |  |  | 8:72111658 | rs117149407 | 0.78% (A) | G | A |  |  |
|  |  |  | 8:72111678 | rs10090382 | 47.50% (C) | T | C |  |  |
|  |  |  | 8:72111710 | rs10103644 | 17.49% (T) | C | T |  |  |
|  |  |  | 8:72111739 | rs10103852 | 47.28% (C) | G | C |  |  |
| ANO1 | 11:69981445-69981895 | 450 | 11:69981591 | rs7947793 | 44.01% (A) | C | A | TATTCCCATGAGTTAAGGAGACGCTGGCGGGCTTGGCCGAG | ACAGCCCAAGACGGCTTCTTTCTTCATCCGGGCTCCTTCAG |
|  |  |  | 11:69981736 | rs7947936 | 46.91% (G) | C | G |  |  |
| OR10G6 | 11:123874598-123875090 | 492 | 11:123874762 | rs11603189 | 21.01% (T) | A | T | CTTCTCAACATTACGACGATTGACGGATCATAAGTAGTGGGA | GGTCAGAGTAATTCGACATTCTACTAAGGTGGACTTGGTTCC |
|  |  |  | 11:123874768 | rs10750254 | 46.65% (C) | T | C |  |  |
|  |  |  | 11:123874858 | NSV |  | A | G |  |  |
|  |  |  | 11:123874903 | NSV |  | A | G |  |  |
|  |  |  | 11:123874968 | rs10750255 | 44.99% (A) | G | A |  |  |
|  |  |  | 11:123874984 | NSV |  | G | T |  |  |
|  |  |  | 11:123875012 | NSV |  | A | G |  |  |
|  |  |  | 11:123875032 | rs538121404 |  | G | T |  |  |
| Chr12:53244 | 12:43253244-43253623 | 379 | 12:43253415 | rs1520832 | 4.49% (T) | T | G | TATGAAAGCTGGACGAGCAGAATTCCCAAACTTTGGTCTGA | TTGCCCACTCACTAGACAGCCGCTCCATCGCAAGGCTCTCTG |
|  |  |  | 12:43253469 | rs7308945 | 31.83% (T) | A | T |  |  |
|  |  |  | 12:43253536 | rs572369996 | 0.02% (A) | G/C | A |  |  |
| HOXC13 | 12:54319518-54320066 | 548 | 12:54319727 | rs894734 | 41.01% (A) | A | G | TATCACATTAAACCGAGCTCAGAGTCTTGAAGCTACATTGC | AGTTCTAATTAATGGAATGCATGTGCCGTTTCCTGGCCAA |
| SGCG | 13:23756241-23756710 | 469 | 13:23756283 | NSV |  | T | G | ATATCAGCTAAGCTTGCAGTTAAAATGCGGAAAATGAAATT | CCTGCAATCCTCATGAGAACGTCGTAGAACTATCTGGCCCT |
|  |  |  | 13:23756363 | rs571976 | 24.22% (G) | G | A |  |  |
|  |  |  | 13:23756594 | rs3794371 | 47.70% (A) | G | A |  |  |
|  |  |  | 13:23756627 | rs3794370 | 49.14% (G) | T | G |  |  |
| Chr13:46674 | 13:112546674-112547008 | 334 | 13:112546833 | rs3903774 | 25.38% (G) | A | G | CACCCAAAGTATGTGGCTCAGCACCGTGCTTATAGGACAC | CATTTGAATAGGGCGTATGTCTTTGGTTGGGCAATGAA |
|  |  |  | 13:112546866 | rs1923740 | 35.32% (C) | C | A |  |  |
| RAD51B | 14:69147989-69148447 | 458 | 14:69148127 | rs1884808 | 40.22% (A) | A | G | TGGAGTGTCTACTCGAGGTGTTCCAAATCAGCGGAGAGTC | GGGAGGTTATTTATACAGTGGCCCAGGACACGATATTACACA |
|  |  |  | 14:69148298 | rs1884807 | 42.33% (A) | G | A |  |  |
| RBFOX1 | 16:5994157-5994565 | 408 | 16:5994224 | rs4786793 | 31.35% (G) | A/C | G | TGGCCATTGATCTAATGACCTCAACGATGTGTGAACAATG | TCACACAGCTCCGAAGGGACCAACTCGGGATTTGCAGCCA |
|  |  |  | 16:5994311 | rs7202039 | 31.33% (T) | T | C |  |  |
|  |  |  | 16:5994318 | rs7202046 | 31.33% (T) | T | C |  |  |
|  |  |  | 16:5994402 | rs7195248 | 31.35% (G) | G | C |  |  |
|  |  |  | 16:5994525 | rs7196781 | 14.02% (G) | C | G |  |  |
| NUP88 | 17:5295079-5295578 | 499 | 17:5295307 | rs148519577 | 0.30% (T) | C | T | TGAAGTTGCTTGGGACATCCTATCAGTACCATAACCTCAA | GTGTGAGAGGAGGCGGCATCCACATTCGGGGGCAGATGTG |
|  |  |  | 17:5295357 | rs1806236 | 3.13% (C) | T | C |  |  |
|  |  |  | 17:5295371 | rs3026129 | 44.77% (T) | T | C |  |  |
|  |  |  | 17:5295399 | rs1806237 | 44.79% (C) | C | T |  |  |
|  |  |  | 17:5295502 | rs1806238 | 44.79% (C) | C | T |  |  |
|  |  |  | 17:5295528 | rs1806239 | 44.79% (T) | T | C |  |  |
|  |  |  | 17:5295529 | NSV |  | G | T |  |  |
|  |  |  | 17:5295534 | NSV |  | C | G |  |  |
|  |  |  | 17:5295547 | NSV |  | C | G |  |  |
| NSF | 17:44799862-44800319 | 457 | 17:44800046 | rs7224296 | 47.18% (G) | G | A | CTACCTAAAGTTGTGGTGCAGCTTCCCGGCTTAGTATGTAA | TTTTGCAGTCGGTCTCTGTGCTAATTACTATCAGAGGGAG |
|  |  |  | 17:44800110 | rs199454 | 39.12% (G) | G | A |  |  |
|  |  |  | 17:44800121 | rs115851737 | 2.92% (A) | A | G |  |  |
| ZADH2 | 18:72919745-72920249 | 504 | 18:72919995 | NSV |  | C | A | GTCCTTCAGTCCGTGCAAGGGACTTTACGGTACTTTCTAGTA | GTCTGCGTGCTGTCAGTGCAGTGTGACTGTTGTCTGTCC |
|  |  |  | 18:72920041 | NSV |  | G | T |  |  |
|  |  |  | 18:72920065 | rs12969004 | 30.45% (A) | G | A |  |  |
|  |  |  | 18:72920070 | NSV |  | T | A |  |  |
|  |  |  | 18:72920103 | rs1866730 | 46.33% (A) | G | A |  |  |
|  |  |  | 18:72920141 | NSV |  | C | A |  |  |
| Chr20:36542 | 20:22136542-22137048 | 506 | 20:22136709 | rs804539 | 3.12% (G) | G | C | AACAATATGAGGCTCTTGACAGTCATAGTTTAGCCCCCTGG | GCCAAGTCTATATGCTGCCAAGGCTATATGCTGGGCATAA |
|  |  |  | 20:22136786 | rs6047929 | 41.71% (G) | G | A |  |  |
|  |  |  | 20:22136839 | rs6113548 | 42.35% (C) | T | C |  |  |
| Chr20:21389 | 20:39521389-39521826 | 437 | 20:39521581 | rs6129700 | 44.19% (G) | A | G | GGTGAGGACGGAAACTGTAAACTCCCGGGAACATTCTGGC | TGATCAGTCTAGGCGTTGGGTCACCCTTTAGCACCAGGTC |
|  |  |  | 20:39521617 | rs6124288 | 38.00% (C) | T | C |  |  |
| Chr20:66266 | 20:56566266-56566520 | 254 | 20:56566432 | rs6092579 | 43.20% (C) | T | C | GGCAGAGGCGGGCCAGTGCTTGGAGTTAGCATGCCCTCCT | CACCAGCCCTAGGCGGTTAATGACTCGCTCAGCTCCTTAA |
|  |  |  | 20:56566479 | NSV |  | T | C |  |  |
| RIPK4 | 21:43163937-43164483 | 546 | 21:43164232 | rs6092580 | 43.15% (T) | A/G | T | GGGTCAAGAAACTGTCCCCTTGATTCCGTTTAGCTTCTCAC | TGCTGTGCCGATGCGGGGTCTGAGCTCGGTGACCAATAGG |
|  |  |  | 21:43164345 | NSV |  | A | C |  |  |
| SLC19A1 | 21:46958834-46959343 | 509 | 21:46959087 | rs2277790 | 31.73% (A) | A | C | GCAGCAGCACGAAGCACACTCGAGGGCGTGTCTCTTTCA | GCCGGCCCTGTGGCGAGGCACAATTGTCCAACTGTCAGCC |
|  |  |  | 21:46959179 | NSV |  | G | A |  |  |
| SYNE3 | 14:95932757-95932898 | 141 | 14:95932819 | rs9976727 | 48.04% (A) | G | A | CTCAGTCCTACTGGATAAGACCCTCTGCCCAGTAAATAAGCTCCCTGCGTGAGGGTAGGAGC | TGCTGGAGGTAAACGGCTCCCCAAGATGCAT |
|  |  |  | 14:95932844 | rs758271841 |  | C | T |  |  |
| PRUNE2 | 9:79312327-79312837 | 510 | 9:79312409 | rs488165 | 43.89% (A) | G | A | GCCTCCAACCACTCGAGTTGGTCCCACA | TTCCATAGTAAGTGCTTGCAGACTGACAA |
|  |  |  | 9:79312491 | rs489069 | 44.29% (A) | C | A |  |  |
|  |  |  | 9:79312556 | rs10869797 | 33.51% (A) | G | A |  |  |
|  |  |  | 9:79312579 | rs684639 | 44.35% (T) | A | T |  |  |
|  |  |  | 9:79312627 | rs685011 | 44.35% (G) | C | G |  |  |
|  |  |  | 9:79312642 | rs12346312 | 5.45% (A) | G | A |  |  |
| Chr17:55225 | 17:14755225-14755669 | 444 | 17:14755298 | NSV |  | C | A | CTGCTTGGTATGCCGTCATACCTTTGCT | CCCAAACTCTGTCCGGTAATAATTTCCA |
|  |  |  | 17:14755310 | rs11652535 | 38.34% (G) | A | G |  |  |
|  |  |  | 17:14755323 | rs73266033 | 17.07% (T) | C | T |  |  |
|  |  |  | 17:14755471 | rs7211522 | 33.77% (T) | C | T |  |  |
|  |  |  | 17:14755492 | rs11652696 | 33.77% (A) | G | A |  |  |
|  |  |  | 17:14755582 | NSV |  | C | A |  |  |
| RPA3-AS1 | 7: 7856490-7856938 | 448 | 7:7856573 | NSV |  | G | A | TAGCCCTTAATGCCCAGTCCTACTTTCATTC | AATGATGAGTCAACCCGAATCCCCACTTCA |
|  |  |  | 7:7856659 | NSV |  | C | A |  |  |
|  |  |  | 7:7856669 | rs6956438 | 21.79% (G) | G | A |  |  |
|  |  |  | 7:7856715 | NSV |  | T | C |  |  |
|  |  |  | 7:7856810 | rs6943254 | 41.83% (T) | T | G |  |  |
|  |  |  | 7:7856830 | NSV |  | T | C |  |  |
|  |  |  | 7:7856853 | rs6957893 | 31.93% (G) | C | G |  |  |
|  |  |  | 7:7856893 | rs6957911 | 11.98% (C) | C | G |  |  |
|  |  |  | 7:7856893 | rs386709970 |  | CT | GC |  |  |
|  |  |  | 7:7856894 | rs6943429 | 45.69% (T) | T | C |  |  |
| TMEM64 | 8:91777679-91778023 | 344 | 8:91777733 | NSV |  | A | G | AGTTACCATGACTTGGCCGAAGACTATGATTG | CCAGGCATCATACTTGGTACACCATGCATAG |
|  |  |  | 8:91777808 | rs16905310 | 8.35% (G) | A | G |  |  |
|  |  |  | 8:91777822 | NSV |  | G | A |  |  |
|  |  |  | 8:91777848 | rs12542205 | 43.39% (A) | A | G |  |  |
|  |  |  | 8:91777905 | rs12544556 | 43.73% (G) | G | A |  |  |
|  |  |  | 8:91777932 | rs12542223 | 43.39% (A) | A | G |  |  |
|  |  |  | 8:91777937 | rs116969974 | 1.76% (T) | C | T |  |  |
|  |  |  | 8:91777948 | rs13265863 | 43.35% (A) | A | G |  |  |
|  |  |  | 8:91777978 | rs12542788 | 43.43% (T) | T | G |  |  |
| LUC7L2 | 7:139079073-139079540 | 467 | 7:139079207 | rs7795520 | 41.45% (G) | A | G | AGTGAGTTAATCTTACCCCTCTCATGCATC | TACCATGCTAGTTCCGGTTATTTCAGTAC |
|  |  |  | 7:139079385 | rs6467846 | 41.43% (T) | G | T |  |  |
|  |  |  | 7:139079436 | rs6467847 | 39.00% (C) | T | C |  |  |
| LRFN2 | 6:40527450-40527803 | 353 | 6:40527521 | rs1771302 | 7.51% (G) | A | G | CTGGGCATGTCCTACCCTAGCTGCCTACT | CACCCAGGGCTAACCGAGACAATGCAGA |
|  |  |  | 6:40527525 | rs1737679 | 40.62% (G) | G | A |  |  |
|  |  |  | 6:40527634 | rs846505 | 44.37% (A) | A | C |  |  |
|  |  |  | 6:40527687 | NSV |  | C | A |  |  |
|  |  |  | 6:40527703 | rs11757640 | 46.41% (T) | C | T |  |  |
| CCL28 | 5:43367913-43368327 | 414 | 5:43368066 | rs10512827 | 43.33% (G) | A | G | TGGCTATCTTGCCCGCAACTACCAATGTATGT | GTTGCAACTACCAGCCTACCATACCAGA |
|  |  |  | 5:43368162 | rs12517362 | 43.25% (T) | C | T |  |  |
|  |  |  | 5:43368290 | rs10512828 | 42.75% (C) | T | C |  |  |
| Chr16:03046 | 16:5903046-5903508 | 462 | 16:5903190 | rs7204977 | 36.98% (G) | T | G | ATATGTTTACAGATCAGACTAGCCCTAGG | CTGGATGGTCCAGCGATTAGATTACCTG |
|  |  |  | 16:5903294 | rs34376349 | 38.96% (A) | G | A |  |  |
|  |  |  | 16:5903304 | rs11643091 | 12.98% (G) | A | G |  |  |
|  |  |  | 16:5903317 | rs7200256 | 28.55% (G) | A | G |  |  |
|  |  |  | 16:5903353 | rs4238867 | 46.61% (T) | G | T |  |  |
| RP11 | X:80722641-80722979 | 338 | X:80722669 | NSV |  | C | T | TACTTGGTAGATGGGTGAGTTTGATAGACAT | CTTGCCCTTAGGGGCTCTATATAAGCCTGC |
|  |  |  | X:80722677 | NSV |  | C | T |  |  |
|  |  |  | X:80722679 | NSV |  | C | T |  |  |
|  |  |  | X:80722680 | NSV |  | C | T |  |  |
|  |  |  | X:80722700 | NSV |  | C | T |  |  |
|  |  |  | X:80722701 | NSV |  | C | T |  |  |
|  |  |  | X:80722706 | NSV |  | C | T |  |  |
|  |  |  | X:80722707 | NSV |  | C | T |  |  |
|  |  |  | X:80722714 | NSV |  | C | T |  |  |
|  |  |  | X:80722715 | NSV |  | C | T |  |  |
|  |  |  | X:80722722 | NSV |  | C | T |  |  |
|  |  |  | X:80722725 | NSV |  | C | T |  |  |
|  |  |  | X:80722728 | NSV |  | C | T |  |  |
|  |  |  | X:80722730 | rs2806667 | 44.74% (A) | G | A |  |  |
|  |  |  | X:80722736 | NSV |  | C | T |  |  |
|  |  |  | X:80722738 | NSV |  | C | T |  |  |
|  |  |  | X:80722743 | NSV |  | C | T |  |  |
|  |  |  | X:80722753 | NSV |  | C | T |  |  |
|  |  |  | X:80722782 | NSV |  | C | T |  |  |
|  |  |  | X:80722785 | NSV |  | C | T |  |  |
|  |  |  | X:80722786 | NSV |  | C | T |  |  |
|  |  |  | X:80722788 | rs2602614 | 43.71% (G) | T | G |  |  |
|  |  |  | X:80722829 | NSV |  | C | T |  |  |
|  |  |  | X:80722839 | NSV |  | C | T |  |  |
|  |  |  | X:80722848 | NSV |  | C | T |  |  |
|  |  |  | X:80722864 | NSV |  | C | T |  |  |
|  |  |  | X:80722875 | NSV |  | C | T |  |  |
|  |  |  | X:80722876 | NSV |  | C | T |  |  |
|  |  |  | X:80722878 | rs1377509 | 44.74% (G) | T | G |  |  |
|  |  |  | X:80722879 | NSV |  | C | T |  |  |
|  |  |  | X:80722937 | rs1377510 | 44.74% (A) | T | A |  |  |
|  |  |  | X:80722974 | NSV |  | G | A |  |  |
| RP11-84D1 | 1:25343997-25344354 | 357 | 1:25344070 | rs10903124 | 46.63% (C) | T | C | GCCAGCCTGACGGGTCAGAGTGTCATCCCGCATTGGTT | TAGGAAACTTACGAGGCCCCTTGGCTAGGATGCGGCCCCA |
|  |  |  | 1:25344122 | rs10903125 | 46.67% (G) | A | G |  |  |
|  |  |  | 1:25344155 | rs10903126 | 46.71% (G) | A | G |  |  |
|  |  |  | 1:25344196 | rs10903127 | 45.71% (A) | G | A |  |  |
|  |  |  | 1:25344265 | rs11249241 | 45.71% (T) | C | T |  |  |
| PLEKHA6 | 1:204285524-204286080 | 556 | 1:204285669 | rs137934729 | 0.54% (A) | G | A | AGCTGCAGGCCGCTCAGAATAGGGACACGGGAGAAATGCTTT | GTTTAAGGAGTGGATACGAGGAGGCCGCTTTATCTGTCTG |
|  |  |  | 1:204285706 | rs4586035 | 44.49% (A) | G | A |  |  |
|  |  |  | 1:204285759 | rs4558047 | 44.47% (A) | G | A |  |  |
|  |  |  | 1:204285934 | rs4433462 | 44.47% (T) | C | T |  |  |
|  |  |  | 1:204286030 | rs4314945 | 44.55% (G) | A | G |  |  |
|  |  |  | 1:204286046 | rs4313451 | 33.45% (A) | A | G |  |  |
|  |  |  | 1:204286046 | rs386638661 |  | AGA | GGG |  |  |
|  |  |  | 1:204286048 | rs4313452 | 44.59% (G) | A | G |  |  |
|  |  |  | 1:204286048 | rs386638661 |  | AGA | GGG |  |  |
|  |  |  | 1:204286061 | rs6692858 | 33.47% (C) | C | T |  |  |
| AGT | 1:230843366-230843888 | 522 | 1:230843438 | rs3789666 | 32.59% (C) | G | C | GGTTCCCCTTGCACGTTCCCTGCAGGGACCTCGGTGCT | CCTAACTTTGCTGGCCTCAGTTTCCGTCAAAGGAGGCA |
|  |  |  | 1:230843446 | rs3789667 | 32.05% (A) | G | A |  |  |
|  |  |  | 1:230843505 | rs2493131 | 4.77% (T) | C | T |  |  |
|  |  |  | 1:230843557 | rs2493132 | 48.84% (T) | T | C |  |  |
|  |  |  | 1:230843633 | rs3789669 | 34.35% (G) | A | G |  |  |
|  |  |  | 1:230843714 | rs3789670 | 18.03% (T) | C | T |  |  |
|  |  |  | 1:230843800 | rs3789671 | 34.31% (T) | G | T |  |  |
| ADI1 | 2:3507764-3508263 | 499 | 2:3507914 | rs11127434 | 47.34% (A) | A | G | CAGGAATCATCACGACTTACGAAAAACGTTGAGTGAAAGTT | GTGGATGTAAATGGTGTGACTCTGACGTGGAAGTTGAGA |
|  |  |  | 2:3507914 | rs71396997 |  | AT | GC |  |  |
|  |  |  | 2:3507915 | rs11891087 | 47.34% (T) | T | C |  |  |
|  |  |  | 2:3507915 | rs71396997 |  | AT | GC |  |  |
|  |  |  | 2:3507931 | rs11127435 | 47.36% (T) | T | C |  |  |
|  |  |  | 2:3507931 | rs386642521 |  | TTGACATCAGCAGT | CTGACATCAGCAGC |  |  |
|  |  |  | 2:3507944 | rs11127436 | 44.21% (T) | T | C |  |  |
|  |  |  | 2:3507944 | rs386642521 |  | TTGACATCAGCAGT | CTGACATCAGCAGC |  |  |
|  |  |  | 2:3508131 | rs11680305 | 44.37% (A) | A | G |  |  |
| RN7SL63P | 2:65785838-65786166 | 328 | 2:65785911 | rs7598635 | 49.64% (G) | G | A | CTTGAATCAGCAACGTCAGCATCACCGGTGGAGTTGTT | ACCAACAGGTTCAATTGCCAGATGACAGACGCTGTCTGTT |
|  |  |  | 2:65785962 | rs1641469 | 48.78% (A) | G | A |  |  |
|  |  |  | 2:65786067 | rs7598574 | 49.76% (C) | C | T |  |  |
|  |  |  | 2:65786077 | rs7601221 | 49.76% (C) | C | T |  |  |
|  |  |  | 2:65786092 | rs7601228 | 49.70% (C) | C | G |  |  |
|  |  |  | 2:65786113 | NSV |  | G | A |  |  |
|  |  |  | 2:65786144 | rs1420350 | 48.86% (T) | A | T |  |  |
|  |  |  | 2:65786161 | rs7601422 | 49.72% (G) | G | C |  |  |
| RP3-468B3 | 6:33917373-33917905 | 532 | 6:33917462 | rs2499746 | 39.06% (T) | C | T | GTCACATTGATGAGTAAACCCTCCTGTCGGAATCTGAAAGCC | AGGTATGGCCTCGGGTTCGAGGGGCTGCGGCGGGAAGCTC |
|  |  |  | 6:33917641 | rs2495959 |  | A | G |  |  |
|  |  |  | 6:33917666 | rs9394181 | 43.17% (T) | G | T |  |  |
|  |  |  | 6:33917711 | rs10947469 | 47.74% (A) | G | A |  |  |
|  |  |  | 6:33917860 | rs9296104 | 48.46% (A) | A | G |  |  |
| RP11-89M16 | 8:129449870-129450320 | 450 | 8:129449877 | rs10099804 | 31.75% (T) | T | C | TGGAAGCTCTTACACGGTCATTTGAGATAGCAGTCATC | ATTGCCTCTTATGGGTGAGATAATGTGTTACGTGTTTCAC |
|  |  |  | 8:129449877 | rs386729892 |  | TCTTACAC | CCTTACAT |  |  |
|  |  |  | 8:129449884 | rs10112885 | 32.11% (C) | C | T |  |  |
|  |  |  | 8:129449884 | rs386729892 |  | TCTTACAC | CCTTACAT |  |  |
|  |  |  | 8:129449908 | NSV |  | A | T |  |  |
|  |  |  | 8:129449914 | NSV |  | C | T |  |  |
|  |  |  | 8:129450151 | NSV |  | A | G |  |  |
|  |  |  | 8:129450156 | rs4733608 | 39.58% (T) | C | T |  |  |
|  |  |  | 8:129450163 | rs4733609 | 31.53% (G) | G | A |  |  |
|  |  |  | 8:129450225 | rs4733610 | 49.54% (G) | G | T |  |  |
| RN7SKP143 | 10:91965949-91966304 | 355 | 10:91965987 | rs2480266 | 31.95% (G) | A | G | ATGAAATGGCATCCGATCCACAAGGTAATGTATGGGGTAG | ACAAACGCAACATAAAGGCAAAATCGTATACATTGTTTT |
|  |  |  | 10:91966084 | rs2478077 | 32.15% (T) | T | C |  |  |
|  |  |  | 10:91966105 | rs2478078 | 32.61% (T) | T | C |  |  |
|  |  |  | 10:91966211 | rs2249784 | 30.93% (G) | G | A |  |  |
|  |  |  | 10:91966298 | rs2249781 | 30.91% (C) | C | A |  |  |
| ANO2 | 12:5790081-5790404 | 323 | 12:5790112 | rs10849323 | 49.58% (T) | T | C | TATAAGAACCAGCCTCTGCATGACAGACCTGTTGTACAGGG | ATCAGATGGCGGGCAAGCTGTTTTCGGCCCAGACTTTC |
|  |  |  | 12:5790119 | rs10774361 | 48.48% (A) | G | A |  |  |
|  |  |  | 12:5790164 | rs7311472 | 14.82% (G) | A | G |  |  |
|  |  |  | 12:5790185 | rs10735052 | 48.52% (C) | T | C |  |  |
|  |  |  | 12:5790195 | rs10744688 | 48.52% (C) | T | C |  |  |
|  |  |  | 12:5790263 | rs10744689 | 48.44% (A) | C | A |  |  |
| OSBP2 | 22:31256275-31256837 | 562 | 22:31256367 | rs8142410 | 31.49% (A) | G | A | GGGTTGGGGTTTAGAGTGAGCCCTAGACAAGAGGAGGCTCTT | TGCTCCAATTCTGGCAACCGCCATGATGCTAAAGATGGCCT |
|  |  |  | 22:31256450 | rs17820330 | 15.14% (G) | C | G |  |  |
|  |  |  | 22:31256498 | rs5753351 | 39.98% (G) | A | G |  |  |
|  |  |  | 22:31256584 | rs4577391 | 16.51% (A) | A | G |  |  |
|  |  |  | 22:31256605 | rs13056704 | 8.81% (T) | C | T |  |  |
|  |  |  | 22:31256668 | rs5749180 | 39.50% (A) | G | A |  |  |
|  |  |  | 22:31256713 | rs5753352 | 16.73% (C) | C | T |  |  |
|  |  |  | 22:31256733 | rs5997791 | 31.63% (A) | C | A |  |  |
|  |  |  | 22:31256766 | rs5997792 | 31.49% (T) | G | T |  |  |
|  |  |  | 22:31256767 | rs5749181 | 48.62% (C) | T | C |  |  |
| Chr8:8340 | 8:123608340-123608909 | 569 | 8:129449884 | rs10112885 | 32.11% (C) | C | T | CATGGGAATGAACCCAATCAGTATCAAC | ACCAGTGACCAAACGATGCATATGACCC |
|  |  |  | 8:129449884 | rs386729892 |  | TCTTACAC | CCTTACAT |  |  |
|  |  |  | 8:129449877 | rs10099804 | 31.75% (T) | T | C |  |  |
|  |  |  | 8:129449877 | rs386729892 |  | TCTTACAC | CCTTACAT |  |  |
